# IGFBP-3 Regulates Mitochondrial Hyperfusion and Metabolic Activity in Ocular Surface Epithelia during Hyperosmolar Stress

**DOI:** 10.3390/ijms23074066

**Published:** 2022-04-06

**Authors:** Whitney L. Stuard, Melis K. Guner, Danielle M. Robertson

**Affiliations:** Department of Ophthalmology, The University of Texas Southwestern Medical Center, 5323 Harry Hines Blvd, Dallas, TX 75390, USA; whitney.stuard@utsouthwestern.edu (W.L.S.); meliskabaalioglu@hotmail.com (M.K.G.)

**Keywords:** cornea, dry eye disease, hyperosmolarity, mitochondria, metabolism, IGFBP-3

## Abstract

In the eye, hyperosmolarity of the precorneal tear film triggers inflammation and the development of dry eye disease (DED), a highly prevalent condition that causes depression and disability in severe forms. A member of the insulin-like growth factor (IGF) family, the IGF binding protein-3 (IGFBP-3), is a pleiotropic protein with known roles in growth downregulation and survival. IGFBP-3 exerts these effects by blocking IGF-1 activation of the type 1 IGF-receptor (IGF-1R). Here, we examined a new IGF-independent role for IGFBP-3 in the regulation of mitochondrial and metabolic activity in ocular surface epithelial cells subject to hyperosmolar stress and in a mouse model of DED. We found that hyperosmolar stress decreased IGFBP-3 expression in vitro and in vivo. Treatment with exogenous IGFBP-3 induced an early, transient shift in IGF-1R to mitochondria, followed by IGFBP-3 nuclear accumulation. IGFBP-3 nuclear accumulation increased protein translation, blocked the hyperosmolar-mediated decrease in oxidative phosphorylation through the induction of mitochondrial hyperfusion, and restored corneal health in vivo. These data indicate that IGFBP-3 acts a stress response protein in ocular surface epithelia subject to hyperosmolar stress. These findings may lead to the development of first-in-class therapeutics to treat eye diseases with underlying mitochondrial dysfunction.

## 1. Introduction

Dry eye disease (DED), as defined in the recent Dry Eye Workshop II (DEWS II) report, “is a multifactorial disease of the ocular surface characterized by a loss of homeostasis of the tear film, accompanied by ocular symptoms, in which tear film instability and hyperosmolarity, ocular surface inflammation and damage, and neurosensory abnormalities play etiological roles” [1]. This chronic and often painful disease affects an estimated 16.4 million Americans alone, increases in prevalence with age, and disproportionately affects females more than males [2,3]. DED is the leading reason that people in the United States seek help from an eye care physician [4]. DED is associated with a significant financial, psychological, and emotional burden due to time lost from work, the high cost of care, chronic pain, and in advanced disease, depression and disability. Moreover, the impact of DED in patients’ day-to-day lives is shown to be comparable to that of moderate-to-severe angina [5]. With no effective treatments or cure, DED creates an enormous public health burden and substantially decreases the overall quality of life in those afflicted.

The underlying pathobiology of DED is linked to hyperosmolarity of the precorneal tear film in both major subtypes: aqueous-deficient DED and evaporative DED [6,7,8]. In aqueous-deficient DED, the lacrimal gland is unable to produce enough aqueous to adequately bathe the surface of the corneal and conjunctival epithelium [6]. In evaporative DED, there is often sufficient aqueous production. Instead, tear film evaporation occurs due to a reduction and alteration in the composition of lipids being produced and released from the meibomian glands that reside in the upper and lower eyelids [6,9]. Both aqueous-deficient and evaporative DED result in an increase in tear osmolarity. This triggers a complex chain of inflammatory events that includes the activation of Toll-like receptors and mitogen-activated protein kinase (MAPK) pathways [10,11,12,13,14,15,16,17]. Dysfunctional aquaporin 5 (AQP5) can also impair water channels present in corneal epithelial cells, leading to the activation of c-Jun N-terminal kinase (JNK1/2) MAPK and an increase in proinflammatory cytokine expression [18]. Similarly, the transient receptor potential vanilloid channel type 1 (TRPV1) signaling pathway mediates the release of inflammatory cytokines (IL-6 and IL-8) through EGFR transactivation, MAPK, and NF-κB stimulation in corneal epithelial cells exposed to hyperosmolar stress [19]. Together, these hyperosmolar induced cascades of pro-inflammatory cytokines and chemokines, along with matrix metalloproteinases that degrade tissue, result in increased ocular surface damage [20,21,22,23,24,25,26,27].

The ocular surface is formed by two contiguous epithelia: the corneal and conjunctival epithelium. In recent years, hyperosmolarity has been linked to a variety of mitochondrial changes in both corneal and conjunctival epithelial cells, including oxidative stress, mitochondrial DNA damage and apoptosis [23,24,28,29,30]. This hyperosmolar-mediated increase in apoptosis is associated with a corresponding loss of barrier function, which is essential to protect the cornea from environmental insults, sheer stress, and pathogen invasion [29,31]. Hyperosmolarity has also been shown to increase oxidative stress and exert negative effects on mitochondria in other non-ocular epithelial cells, such as intestinal and bronchial epithelia [32,33,34,35,36,37]. This includes the upregulation of proinflammatory mediators in these cell types. Thus, in addition to ocular surface disease, hyperosmolar stress is a major underlying factor in the pathophysiology of many inflammatory lung and bowel disorders.

The insulin-like growth factor (IGF) family consists of ligands, cell surface receptors, and insulin-like binding proteins (IGFBPs) that function to mediate cell growth and survival throughout the body. Of the six known binding proteins, IGFBP-3 is the most abundant binding protein in circulation where it functions to block IGF-1-mediated activation of the type 1 IGF receptor [38]. IGFBP-3 is also produced locally in many tissues and is present within the human precorneal tear film [39,40]. We have previously shown that IGFBP-3 is downregulated in response to hyperosmolar stress in corneal epithelial cells [41]. We further identified a novel role for IGFPB-3 in mitochondrial homeostasis and quality control in corneal and conjunctival epithelial cells [42]. These intricate networks are essential for optimal mitochondrial function and epithelial cell health. In contrast, mitochondrial failure or dysfunction triggers degenerative cellular processes, such as those seen in DED. In this study, we interrogated the role of IGFBP-3 in mediating mitochondrial morphology and metabolic activity in corneal and conjunctival epithelial cells exposed to hyperosmolar stress. Using a mouse model of aqueous-deficient DED, we further investigated the effect of topical treatment with exogenous IGFBP-3 on the ocular surface in vivo.

## 2. Results

### 2.1. IGFBP-3 Expression Levels Parallel Mitochondrial Respiration under Increasing Hyperosmolar Stress

**Figure 1:**
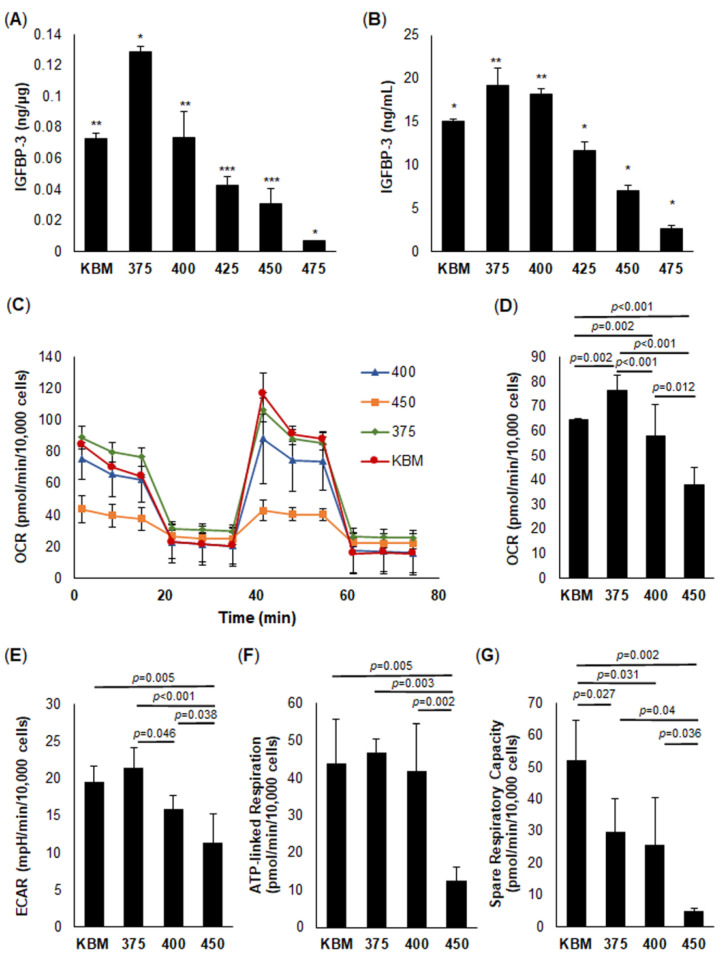
Insulin-like growth factor binding protein-3 (IGFBP-3) levels parallel changes in mitochondrial respiration under increasing hyperosmolar stress. hTCEpi cells were treated with increasing osmolarity levels in keratinocyte basal media (KBM) for 24 h. Intra- and extracellular levels of IGFBP-3 were quantified using ELISA. (**A**) Intracellular IGFBP-3 expression was significantly increased in 375 mOsm, followed by a concentration-dependent decrease in IGFBP-3 (* *p* < 0.001, significantly different from all other groups; ** *p* < 0.001, *** *p* < 0.001 significantly different from all other groups except each other). (**B**) Extracellular levels of IGFBP-3 showed a similar trend (* *p* < 0.001, significantly different from all other groups; ** *p* < 0.001, significantly different from all other groups except each other). (**C**–**G**) Seahorse metabolic flux analysis. (**C**) Oxygen consumption rate (OCR) plotted as a function of time. Arrows indicate timepoints at which oligomycin (O), FCCP (**F**), and rotenone/antimycin A (R/A) were added. (**D**) OCR was increased at 375 mOsm compared to control and sequentially decreased with increasing levels of salt. (**E**) Extracellular acidification rate (ECAR) was not significantly increased at 375 mOsm, but was decreased at higher osmolarity. (**F**) ATP-linked respiration decreased at the highest osmolarity tested (450 mOsm). (**F**) Spare respiratory capacity decreased in all conditions with increased osmolarity. Data are represented as mean ± standard deviation from a single experiment that was independently repeated three times, one-way ANOVA, and SNK multiple comparison test).

Previous studies in our lab have shown that a high level of osmolar stress (450 mOsm) decreases intra- and extracellular levels of IGFBP-3. This decrease was associated with a reduction in metabolic activity. To further examine the relationship between hyperosmolarity and expression levels of IGFBP-3, cells were cultured in KBM with increasing levels of osmolar stress (375, 400, 425, 450, and 475 mOsm) for 24 h. Intracellular and extracellular levels of IGFBP-3 were then measured using ELISA (Figure 1A,B, respectively). While the intracellular expression of IGFBP-3 was much lower than the extracellular secreted protein, both showed an initial increase in IGFBP-3, peaking at 375 mOsm, followed by a salt-concentration-dependent decline. Consistent with this, HCjECs and HCECs all showed a similar expression pattern (Supplementary Figure S1A–D).

Next, we tested the effects of increasing osmolar stress on mitochondrial respiration using a mito-stress test. Interestingly, the measured changes in OCR and ECAR paralleled IGFBP-3 expression levels, again peaking at 375 mOsm (*p* = 0.002, Figure 1C,D). As shown in Figure 1D, the hyperosmolar-mediated decrease in OCR at 400 mOsm was significantly lower than isotonic KBM or 375 mOsm (*p* < 0.001, compared to 375 mOsm; *p* = 0.002 compared to the isotonic control). OCR was further reduced at 450 mOsm (*p* < 0.001 compared to 375 mOsm and the isotonic control, *p* = 0.012 compared to 400 mOsm). Since OCR is a combination of basal mitochondrial respiration and non-mitochondrial respiration, the subsequent analysis of basal respiration also showed a decrease at 450 mOsm (Supplementary Figure S2A, *p* = 0.002, *p* = 0.003, *p* = 0.008, 450 compared to KBM, 375 mOsm, and 400 mOsm, respectively). There were no differences in non-mitochondrial oxygen consumption between groups (Supplementary Figure S2B). Unlike OCR, there was no significant difference in ECAR between 375 mOsm and the isotonic control; however, as the osmolarity increased to 400 mOsm, ECAR dropped below the isotonic control (Figure 1E, *p* = 0.046). ECAR continued to decrease at 450 mOsm and was significantly reduced compared to all other conditions (Figure 1E, *p* = 0.005, *p* < 0.001, and *p* = 0.038 compared to KBM, 375 mOsm and 400 mOsm, respectively) To analyze the components of the electron transport chain, hTCEpi cells were treated with 1.0 μM oligomycin to block the function of ATP synthase and quantify ATP-linked respiration. At low levels of hyperosmolar stress, there were no detectable changes in ATP-linked respiration (Figure 1F). ATP-linked respiration was only reduced at the highest osmolarity tested (450 mOsm, *p* = 0.005, *p* = 0.003, *p* = 0.002 compared to KBM, 375 mOsm, and 400 mOsm, respectively)**.** In contrast, there was no change in proton leak (Supplementary Figure S2C). To determine the effect of hyperosmolarity on respiratory capacity, hTCEpi cells were treated with an electron transport chain uncoupler and trifluoromethoxy carbonylcyanide phenylhydrazone (FCCP), which allows for the maximal flow of electrons through the electron transport chain. As shown in Figure 1G, the spare respiratory capacity was decreased at all levels of osmolar stress compared to the isotonic control (*p* = 0.027, 375 mOsm compared to control; *p* = 0.031, 400 mOsm compared to control; *p* = 0.002, 450 mOsm compared to control). The maximal respiration showed a similar pattern (Supplementary Figure S2D, *p* = 0.002, *p* = 0.004, *p* = 0.004, 450 compared to KBM, 375, and 400, respectively). In addition, there was a small but significant decrease in coupling efficiency at 450 mOsm compared to the control and all other test groups (Supplementary Figure S2E, *p* = 0.013, *p* = 0.003, *p* = 0.004, compared to KBM, 375, and 400, respectively). Taken together, these data suggest that IGFBP-3 expression is required for the maintenance of mitochondrial respiration and respiratory capacity in cells exposed to hyperosmolar stress.

### 2.2. IGFBP-3 Does Not Impact Cell Growth or Cell Cycle in Cells Exposed to Hyperosmolar Stress

**Figure 2:**
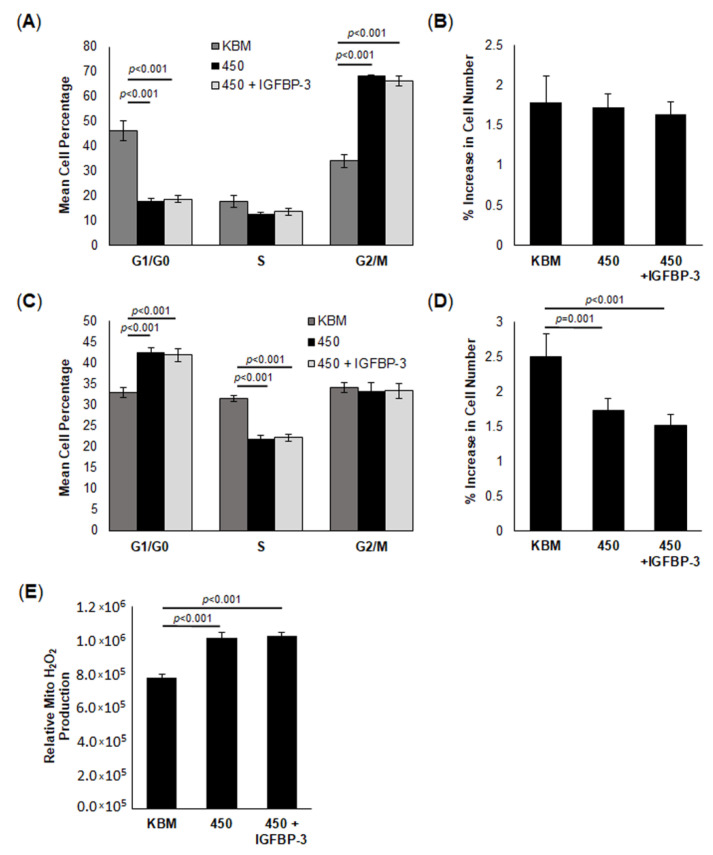
IGFBP-3 does not impact cell growth or cell cycle in cells exposed to hyperosmolar stress. hTCEpi cells were cultured in 450 mOsm KBM with or without recombinant human (rh)IGFBP-3. Cell number and cell cycle were assessed at 6 and 24 h. (**A**) At 6 h, cells in 450 mOsm were arrested in G2/M. Treatment with rhIGFBP-3 did not alter growth arrest. (**B**) At 6 h, there was no significant difference in cell number amongst groups. (**C**) At 24 h, cells in hyperosmolar culture were arrested in G1/G0. Again, rhIGFBP-3 failed to alter cell cycle. (**D**) At 24 h, cell number was decreased in 450 mOsm KBM. rhIGFBP-3 had no effect. (**E**) Mitochondrial H_2_O_2_ production was measured using an Amplex Red fluorescence assay. Hyperosmolar stress triggered an increase in mitochondrial H_2_O_2,_ while rhIGFBP-3 had no effect. Graphs are representative of a single experiment repeated 3 times. Data are represented as mean ± standard deviation, one-way ANOVA and SNK multiple comparison test.

In our prior studies, we found that IGFBP-3 arrests the cell cycle in G2/M, inhibiting cell growth during the log growth phase. In subsequent work, we reported that increasing levels of hyperosmolar stress similarly triggered cell cycle arrest and reduced cellular growth. To determine whether IGFBP-3 impacts cell growth in cells exposed to hyperosmolar stress, hTCEpi cells were cultured in KBM under isotonic or hyperosmolar conditions (450 mOsm), with or without 500 ng/mL rhIGFBP-3 for six or twenty-four hours. Consistent with our prior work, six hours of hyperosmolar culture arrested hTCEpi cells in the G2/M phase (Figure 2A, *p* < 0.001) [41]. Unlike the beneficial effects on mitochondrial respiration, treatment with rhIGFBP-3 did not affect cell cycle or cell number (Figure 2A,B). As expected, after twenty-four hours of hyperosmolar culture, cells were now arrested in the G1/G0 phase (Figure 2C, *p* < 0.001). Again, co-treatment with IGFBP-3 failed to restore the hyperosmolar-mediated G1/G0 arrest. At this 24 h time point, cell number was decreased and remained unchanged by treatment with rhIGFBP-3 (Figure 2D, *p* = 0.001, 450 compared to KBM; *p* < 0.001, 450 + IGFBP-3 compared to KBM). The reduced cell number in hTCEpi cells exposed to hyperosmolar culture was associated with an increase in ROS production in these same treatment groups (Figure 2E, *p* < 0.001 for 450 and 450 with rhIGFBP-3 compared to KBM). Taken together, these data show that in cells subject to hyperosmolar stress with high levels of ROS, IGFBP-3 no longer functions as a growth inhibitory protein.

### 2.3. IGFBP-3 Drives IGF-1R Translocation to the Mitochondria during Hyperosmolar Stress 

**Figure 3:**
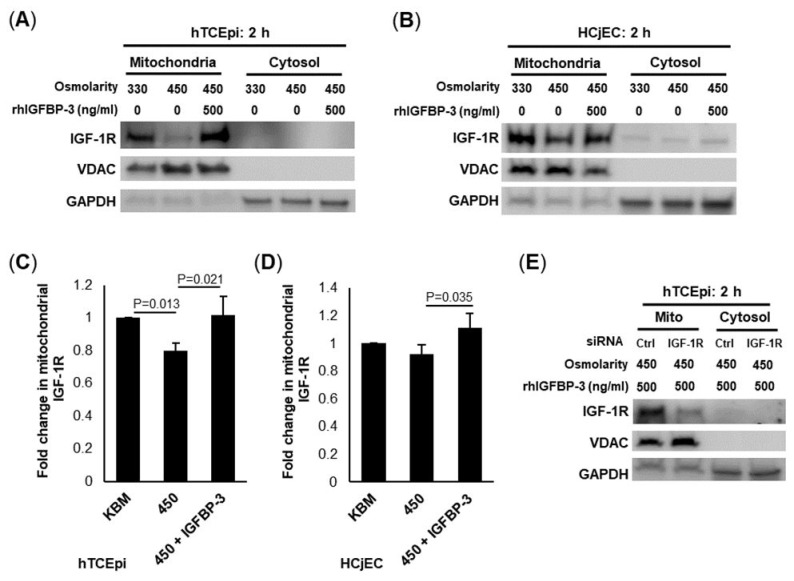
IGFBP-3 promotes IGF-1 receptor (IGF-1R) accumulation in mitochondria during hyperosmolar stress. hTCEpi cells were cultured in isotonic or 450 mOsm KBM for 24 h. Additional cells in 450 mOsm KBM were co-treated with 500 ng/mL rhIGFBP-3. hTCEpi cells and HCjECs were separated into cytosolic and mitochondrial fractions and immunoblotted for IGF-1R. VDAC was used as a mitochondrial control and GAPDH for a cytosolic control. (**A**) In hTCEpi cells, IGF-1R expression was decreased in hyperosmolar conditions. This decrease was blocked by co-treatment with rhIGFBP-3. Similar changes were seen in (**B**) HCjECs. (**C**) Quantification of mitochondrial IGF-1R in hTCEpi cells, (**D**) HCjECs. (**E**) hTCEpi cells were transfected with siRNA oligonucleotides targeting IGF-1R; non-targeting oligonucleotides were used as a control. Immunoblotting of mitochondrial fractions showed a decrease in mitochondrial IGF-1R, confirming specificity of the antibody to the mitochondrial localized receptor. Data are presented as mean ± standard deviation from 3 representative experiments and analyzed using a one-way ANOVA and SNK multiple comparison test.

Our prior studies found that IGF-1R localizes to the mitochondria in corneal epithelial cells, where it interacts with the voltage-dependent anion channel, VDAC [43]. To investigate a potential role for IGFBP-3 in mitochondrial trafficking of IGF-1R, we used a mitochondrial fractionation assay followed by immunoblotting (Figure 3A,B). To accomplish this, hTCEpi cells and HCjECs were cultured in KBM under isotonic or hyperosmolar conditions (450 mOsm), with or without 500 ng/mL rhIGFBP-3 for two hours. Immunoblotting showed that hyperosmolar stress decreased mitochondrial levels of IGF-1R (*p* = 0.013, Figure 3A,C), while co-treatment with rhIGFBP-3 maintained mitochondrial levels of IGF-1R at isotonic levels (*p* = 0.021, compared to 450 mOsm). The outer mitochondrial membrane protein, VDAC, was used as a loading control to normalize mitochondrial levels of IGF-1R. GAPDH was used to confirm the absence of cross-contamination. A similar trend was also seen in HCjECs (*p* = 0.035, Figure 3B,D). As shown in Supplementary Figure S3A,B, IGFBP-3 knockdown slightly decreased mitochondrial IGF-1R similar to hyperosmolar stress, whereas treatment with rhIGFBP-3 increased mitochondrial IGF-1R (*p* = 0.016, compared to the siRNA IGFBP-3 knockdown). To ensure that mitochondrial IGF-1R was not an artifact due to antibody non-specificity, cells were transfected with siRNA oligonucleotides targeting IGF-1R (Figure 3E). Collectively, these data show that IGFBP-3 is important for the mitochondrial targeting of IGF-1R in cells under hyperosmolar stress.

**Figure 4:**
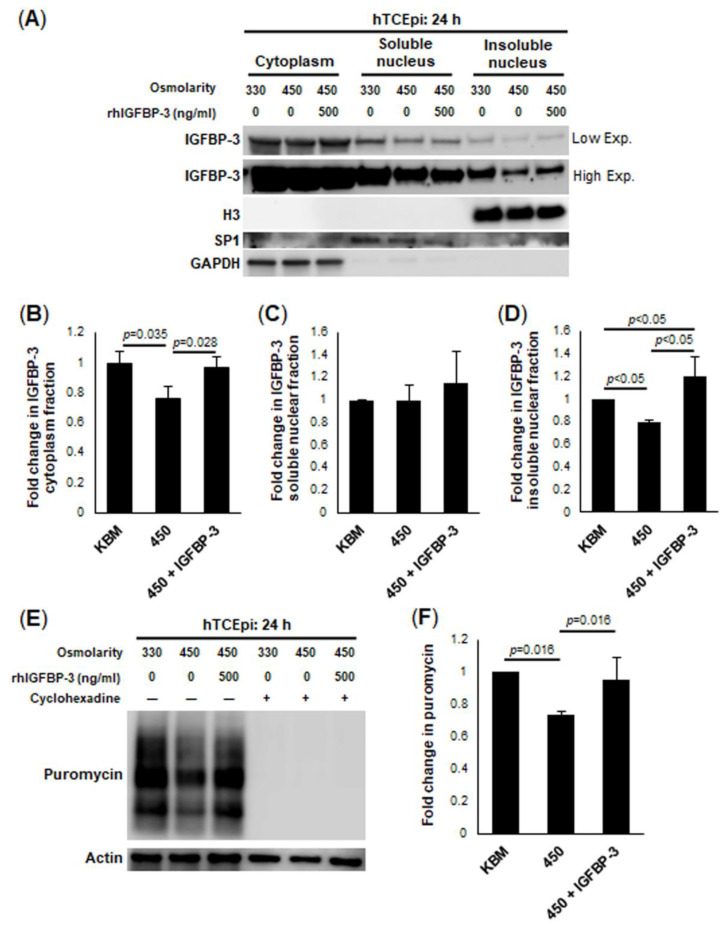
Treatment with exogenous IGFBP-3 drives IGFBP-3 accumulation in the insoluble nucleus during hyperosmolar stress. hTCEpi cells were treated with isotonic or 450 mOsm KBM for 24 h. Additional cells in 450 mOsm KBM were co-treated with 500 ng/mL rhIGFBP-3. (**A**) Cell lysates were separated into cytoplasmic, soluble nuclear and insoluble nuclear fractions. GAPDH was used as a loading control for the cytosolic fraction, SP1, for the soluble nuclear fraction, and H3 for the insoluble nuclear fraction. There was a decrease in IGFBP-3 in 450 mOsm without rhIGFBP-3 in the cytoplasm and insoluble nuclear fraction compared to control. Co-treatment with rhIGFBP-3 blocked the hyperosmolar-mediated decrease. Low exposure of the membrane was used to visualize IGFBP-3 in the cytoplasm and high exposure was used to visualize IGFBP-3 in the insoluble nucleus. (**B**) Quantitative data for IGFBP-3 in the cytoplasm, (**C**) soluble nucleus, and (**D**) insoluble nucleus. (**E**) A SUnSET puromycin assay was used to examine global protein translation. β-actin was used as a loading control. Similar to the findings above, there was a decrease in protein translation in cells cultured in 450 mOsm KBM that was not evident in cells co-treated with rhIGFBP-3. Cyclohexadine was used to confirm that only newly translated protein was observed. (**F**) Quantification of puromycin bands from Western blot. Data are presented as mean ± standard deviation from 3 representative experiments and analyzed using a one-way ANOVA and SNK multiple comparison test.

While IGFBP-3 induced IGF-1R translocation to the mitochondria, we have previously shown that IGFBP-3 itself localizes to the nucleus of corneal epithelial cells [44]. Moreover, following siRNA knockdown of IGFBP-3, there is a small but visible shift in residual IGFBP-3 to the insoluble nucleus. This increase in IGFBP-3 in the insoluble nuclear fractionation was associated with an increase in mitochondrial respiration. To determine whether the downregulation of IGFBP-3 in cells under hyperosmolar stress similarly triggered the residual accumulation of IGFBP-3 in the nucleus, we separated cell lysates into cytosolic, soluble nuclear and insoluble nuclear fractions (Figure 4A–D). Immunoblotting confirmed a decrease in cytoplasmic levels of IGFBP-3 at 450 mOsm (*p* = 0.035 compared to control, Figure 4A,B). This was restored by treatment with rhIGFBP-3 (*p* = 0.028, Figure 4A,B). While there was no change in IGFBP-3 levels in the soluble nucleus (Figure 4A,C), IGFBP-3 decreased at 450 mOsm in the insoluble nucleus (*p* < 0.05, Figure 4A,C). Unlike the cytoplasmic localized protein, however, treatment with rhIGFBP-3 increased the amount of insoluble nuclear IGFBP-3 compared to control levels (*p* < 0.05, 450 + IGFBP-3 compared to 450 and KBM, Figure 4A,D). Indeed, this decrease in IGFBP-3 in the insoluble nucleus in 450 mOsm was associated with our reported decrease in mitochondrial respiration under this same condition (Figure 1D). In contrast, we know that rhIGFBP-3 increases mitochondrial respiration after siRNA knockdown [42]. Using puromycin, we further show that total protein translation is reduced in our hyperosmolar model (*p* = 0.016, Figure 4E,F), and that this hyperosmolar decrease is blocked by the addition of rhIGFBP-3 (*p* = 0.016). Thus, we speculate that in cells with transcriptional downregulation of IGFBP-3, the subsequent accumulation of exogenously applied rhIGFBP-3 in the insoluble nuclear fraction is essential for the transcription of genes involved in oxidative phosphorylation. This would allow the cell to meet the energy demand that is required for adequate mitochondrial function during stress.

### 2.4. IGFBP-3 Promotes Mitochondrial Fusion in Cells Subject to Hyperosmolar Stress

**Figure 5:**
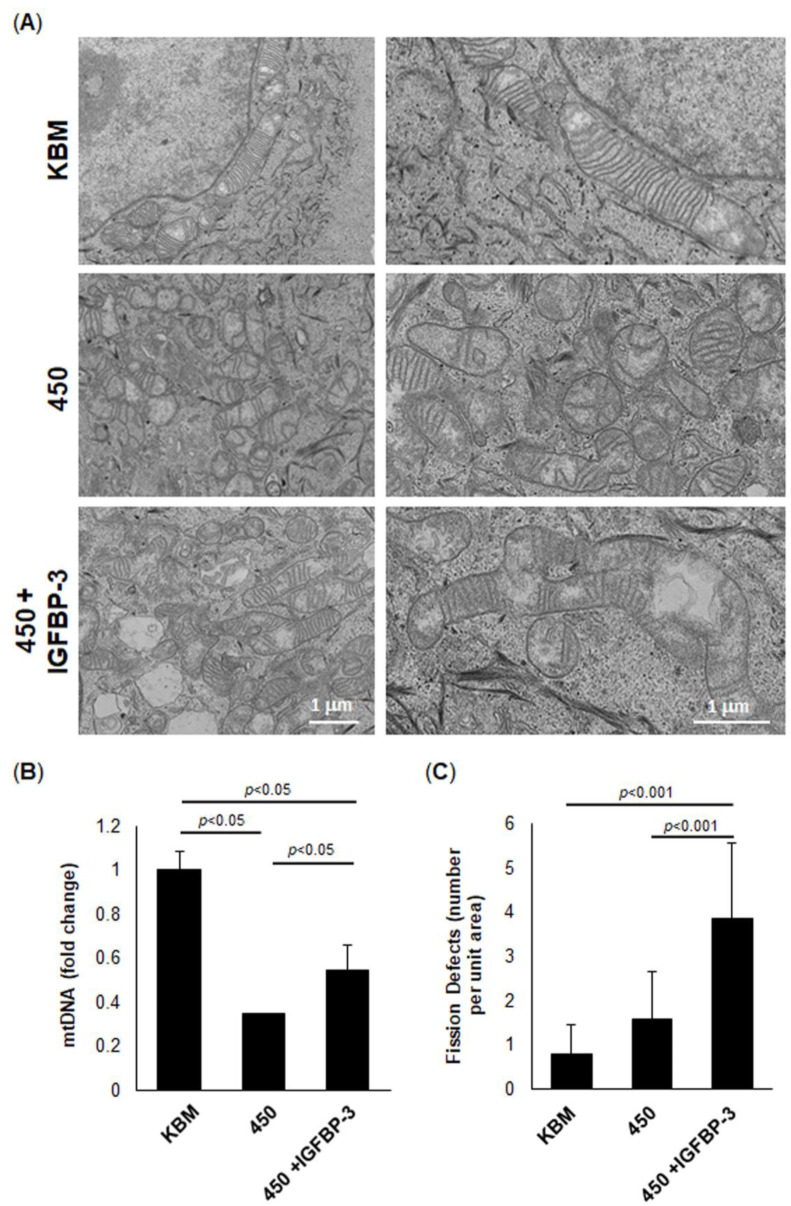
IGFBP-3 induces fission defects in mitochondria exposed to hyperosmolar stress. Cells were cultured in KBM or 450 mOsm KBM with or without 500 ng/mL rhIGFBP-3 for 24 h. (**A**) Transmission electron microscopy was performed to assess mitochondrial ultrastructure. Scale bar: 1 µm. (**B**) mtDNA was quantified using qPCR. Hyperosmolar stress decreased mtDNA (*p* < 0.05), whereas co-treatment with rhIGFBP-3 partially restored mtDNA to basal levels. **(C)** Quantification of fission defects per unit area. Co-treatment with rhIGFBP-3 increased the number of fission defects (*n* = 10 images). Graphs representative of a single experiment that was repeated 3 times. Data are presented as mean ± standard deviation, one-way ANOVA and SNK multiple comparison test.

Our lab has previously shown that IGFBP-3 is an integral protein involved in the regulation of mitochondrial ultrastructure and cristae maintenance [42]. Therefore, we next performed transmission electron microscopy to visualize mitochondrial ultrastructure in hTCEpi cells under hyperosmolar stress with or without rhIGFBP-3. As shown in Figure 5A, there were sparse but morphologically intact mitochondria and cristae in cells during isotonic culture in KBM. Hyperosmolar stress showed an increase in small mitochondria with wavy or missing cristae. The robust increase in small mitochondria was suggestive of an increase in mitochondrial fission and was associated with a decrease in mtDNA (Figure 5B, *p* < 0.05, 450 mOsm compared to KBM). These changes would explain the decrease in mitochondrial respiration in cells exposed to hyperosmolar stress. Co-treatment with rhIGFBP-3 reversed these changes, evidenced by a large population of elongated mitochondria with intact lamellar cristae (Figure 5A). This increase in elongated mitochondria corresponded to an increase in mtDNA compared to hyperosmolar stress without IGFBP-3 (Figure 5B, *p* < 0.05, 450 + IGFBP-3 compared to 450 mOsm and KBM). These elongated mitochondria demonstrated “fission defects”, which we defined as areas where the elongated mitochondria were branching but had not undergone fission (Figure 5C, *p* < 0.001, 450 mOsm compared to either 450 or KBM). Taken together, these data indicate that IGFBP-3 plays an important role in maintaining mitochondrial ultrastructure and homeostasis.

### 2.5. IGFBP-3 Maintains Expression of Key Fusion Proteins That Are Decreased during Hyperosmolar Stress 

**Figure 6:**
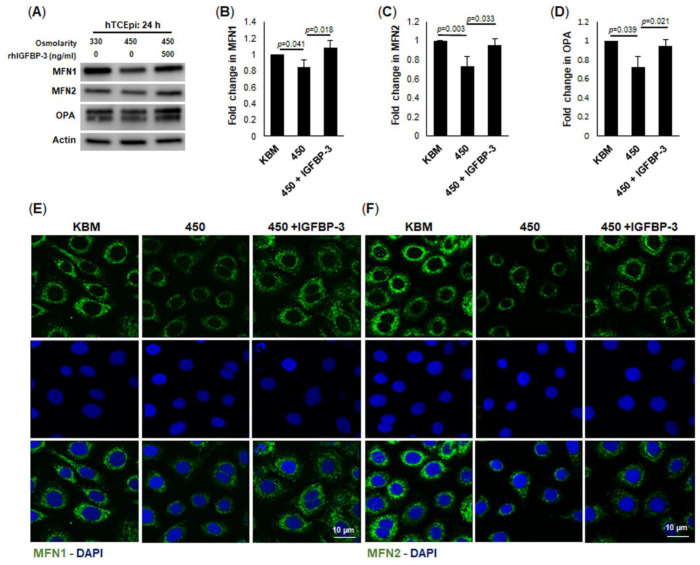
IGFBP-3 mediates fission defects in mitochondria under hyperosmolar stress. hTCEpi cells were treated with isotonic KBM or 450 mOsm KBM for 24 h. An additional set of cells in 450 mOsm KBM were co-treated with 500 ng/mL rhIGFBP-3. (**A**) Immunoblotting for MFN1, MFN2 and OPA. Expression of all 3 proteins decreased in 450 mOsm culture. These decreases were blunted by co-treatment with rhIGFBP-3. β-actin was used as a loading control. (**B**–**D**) Quantification of MFN1 (**B**), MFN2 (**C**), and OPA1 (**D**). (**E**,**F**) Immunofluorescent staining for MFN1 (green, **E**), and MFN2 (green, **F**), nuclei counterstained with DAPI (blue). Staining also showed a decrease in the expression of both proteins in 450 mOsm culture. This decrease was not evident in cells cultured in 450 mOsm treated with rhIGFBP-3. Scale bar: 10 µm. Images and blots are representative of 3 repeated experiments. Graphical data are presented as mean ± standard deviation, one-way ANOVA and SNK multiple comparison test.

Fission and fusion are dynamic processes that function to maintain mitochondrial homeostasis. To further examine changes in mitochondrial ultrastructure, we examined key proteins involved in mitochondrial fusion. Cells were treated with 450 mOsm KBM with or without rhIGFBP-3. During hyperosmolar stress, the fusion proteins MFN1, MFN2, and OPA were decreased compared to control cells, demonstrating a shift towards increased mitochondrial fission (Figure 6A–D). Consistent with our immunoblotting data, decreases in MFN1 and MFN2 were also visible by immunofluorescence (Figure 6E,F). This decrease in fusion proteins was consistent with the numerous small, dense mitochondria visualized by transmission electron microscopy. Co-treatment with rhIGFBP-3 maintained the expression of these fusion proteins at isotonic levels. Collectively, these data suggest that uptake of extracellular IGFBP-3 mediates mitochondrial fusion.

### 2.6. IGFBP-3 Is Decreased in the Corneal Epithelium of Mice with Aqueous-Deficient DED

**Figure 7:**
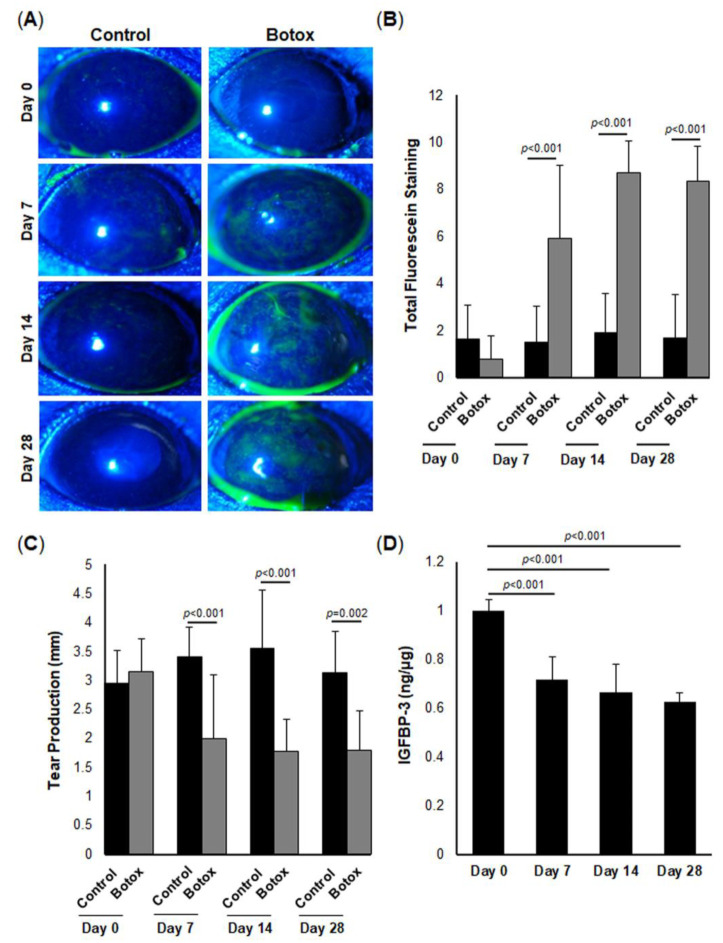
IGFBP-3 is decreased in the murine corneal epithelium in aqueous-deficient dry eye disease (DED). The lacrimal gland was injected with botulinum toxin or a vehicle control. Slit-lamp examination and phenol red thread test were performed at baseline, and then on days 7, 14, and 28 post-injection. (**A**) Slit-lamp examination showing corneal staining with fluorescein in mice using cobalt blue light. (**B**) Total corneal staining grade of all five quadrants (scale 0–3). No difference was evident at baseline (*n* = 72). At all of the time points post-injection (*n* = 58), the Botox-treated group exhibited more corneal staining compared to the vehicle control. (**C**) In the same group of mice, a phenol red thread test was used to measure tear production (mm). There was no difference between the test and control groups at day 0. Tear production was decreased 7 days post-injection and remained low at 28 days. (**D**) The expression level of IGFBP-3 in the mouse corneal epithelium was quantified using ELISA. By day 7, there was a measurable decrease in IGFBP-3 in the corneal epithelium that persisted at day 28 (*n* = 9 per group, 3 corneas from 3 mice were pooled and used as biological replicates and tested on subsequent days). All pooled samples were run in triplicate. Data are presented as mean ± standard deviation, one-way ANOVA and SNK multiple comparison test.

To further investigate the effects of hyperosmolarity on corneal epithelial cells, we created an aqueous-deficient DED mouse model using botulinum toxin B to reduce tear secretion. To verify this model, we performed routine clinical dry eye tests including fluorescein staining for corneal surface damage and a phenol red thread test for tear production. Fluorescein staining was observed via a slit-lamp examination (Figure 7A). Seven days post-Botox, there was a significant increase in corneal fluorescein staining compared to control (*p* < 0.01, Figure 7B). Corneal staining persisted at 14 and 28 days of disease (*p* < 0.001 compared to control). The corneal area was then subdivided into five quadrants (Supplementary Figure S4A). Corneal staining was not restricted to any one region, but was significantly decreased in all five quadrants from day 7, all the way up to 28 days (*p* < 0.001, Supplementary Figure S4B–F). Consistent with corneal staining, the phenol read thread test also showed a significant reduction in aqueous tear production at day 7 in the Botox group compared to control (*p* < 0.001, Figure 7C). Similar to staining, tear production remained decreased at 14 and 28 days (*p* < 0.001, *p* = 0.002, 14 and 28 days, respectively, Figure 7C). These data confirm the induction of aqueous-deficient DED in this model.

**Figure 8:**
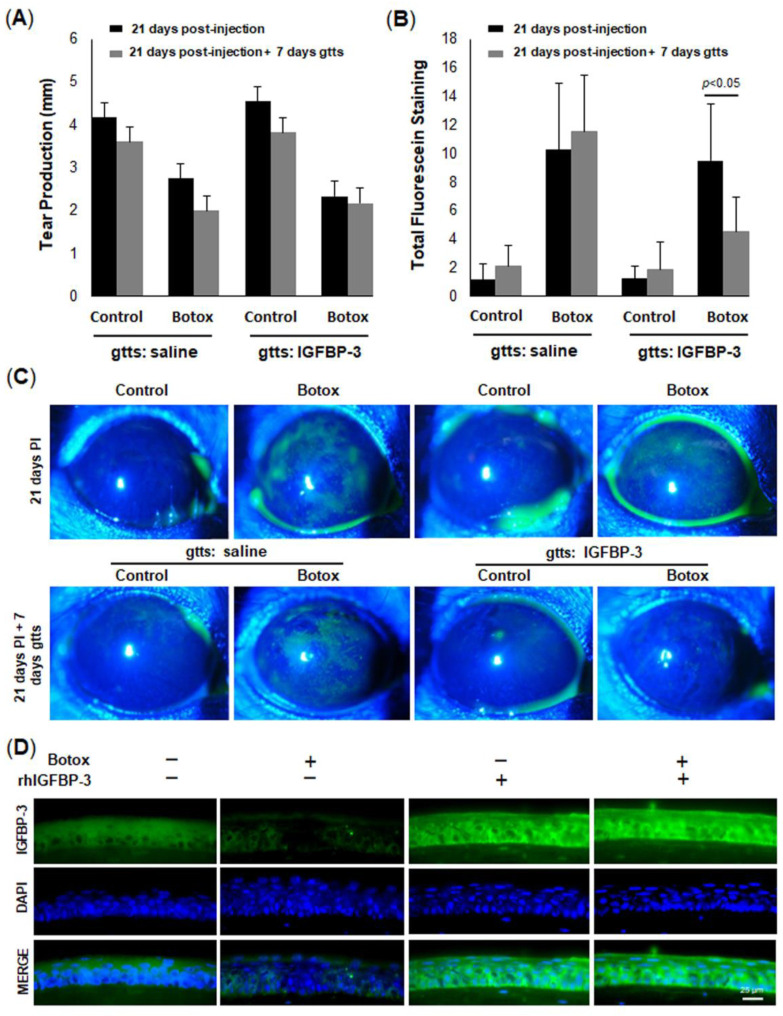
Topical rhIGFBP-3 decreases corneal staining in a mouse with aqueous-deficient DED. The lacrimal gland was injected with botulinum toxin or a vehicle control. After 21 days, mice were treated topically with 10 μL of sterile saline with or without 500 ng/mL rhIGFBP-3 twice a day every other day for 7 days. (**A**) After seven days of dosing, aqueous tear production by the lacrimal gland was unchanged in the treatment and control groups (*n* = 4 mice per group). (**B**) Total corneal staining was decreased in the Botox group treated with rhIGFBP-3 compared to saline (*n* = 8 mice per group). (**C**) Representative slit-lamp images of corneal fluorescein staining in mice. (**D**) Immunostaining of corneal cryosections for IGFBP-3 (green) and DAPI (blue). Scale bar: 25 μm. Graphical data are presented as mean ± standard deviation, one-way ANOVA and SNK multiple comparison test. Slit lamp images representative of 8 mice per group. Immunostaining images representative of 3 mice per group. Gtts: eye drops; PI: post-injection.

**Figure 9:**
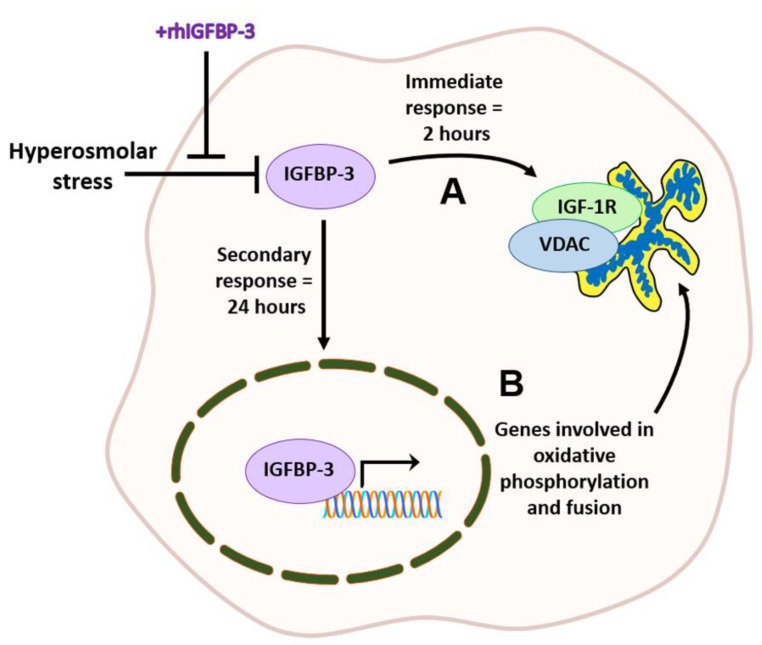
IGFBP-3 mediates the cellular stress response to hyperosmolarity in ocular surface epithelia. (**A**) In the first few hours after the induction of hyperosmolar stress, IGFBP-3 is decreased to very low levels. Co-treatment with exogenous IGFBP-3 induces the translocation of IGF-1R to mitochondria (immediate response). (**B**) Hyperosmolar stress decreases IGFBP-3 in the nucleus, which is associated with a decrease in mitochondrial respiration and fusion. Co-treatment with exogenous IGFBP-3 shifts IGFBP-3 into the nucleus, blocking the hyperosmolar-mediated decrease in protein translation, mitochondrial respiration and fusion.

We next sought to examine the effect of DED on IGFBP-3 expression levels in the mouse corneal epithelium. The corneal epithelium was harvested from mice at 0 (baseline), 7, 14, and 28 days. Similar to the effects of hyperosmolar stress on cells in culture, IGFBP-3 expression was decreased in the mouse corneal epithelium compared to control at all time points tested (Figure 7D, *p* < 0.001 compared to baseline). To further examine the effect of IGFBP-3 on corneal health, IGFBP-3 was topically applied to mouse corneas twice a day every other day for seven days [41]. Treatment was initiated after 21 days of DED. As expected, the topical administration of IGFBP-3 had no effect on tear production (Figure 8A); however, corneal staining was significantly reduced (Figure 8B,C, *p* < 0.05 Botox compared to control). Immunofluorescent staining of cryostat sectioned mouse corneas confirmed uptake of exogenous IGFBP-3 in the corneal epithelium (Figure 8D). When divided into quadrants, treatment with exogenous IGFBP-3 reduced corneal staining in all five quadrants (Supplementary Figure S5A–E). A schematic summarizing these findings is shown in Figure 9.

## 3. Discussion

We recently reported that IGFBP-3 functions to inhibit mitophagy and promote mitochondrial respiration in corneal and conjunctival epithelial cells [42]. We have further shown that IGFBP-3 is decreased during hyperosmolar stress, and thus may play a role in stabilizing mitochondria [41]. The mechanism by which this occurs is unclear. In the present study, we sought to interrogate the role of IGFBP-3 in mitochondrial and metabolic activity in response to hyperosmolar stress in vitro and in a mouse model of aqueous-deficient dry eye in vivo. Importantly, we found that IGFBP-3 expression and secretion are directly related to changes in mitochondrial respiration in response to increasing osmolarity. Specifically, we found that small increases in hyperosmolarity drive an increase in IGFBP-3 expression and secretion, along with increased mitochondrial respiration. This finding is consistent with our hyperglycemia model where cultures in elevated glucose also led to an increase in IGFBP-3 [39]. Interestingly, however, as osmolarity continued to increase, IGFBP-3 levels decreased in a concentration-dependent manner. This was reflected by a parallel decrease in respiration. 

In contrast to respiration, spare respiratory capacity was decreased compared to the isotonic condition at all concentrations tested. Since spare respiratory capacity is a measure of a cell’s energy reserves, even low levels of hyperosmolarity are sufficient to disrupt the capacity of a cell to undergo metabolic adaptation and survival [45]. We speculate that the increase in IGFBP-3 at the lowest osmolar stress tested (375 mOsm) was due to the cell trying to draw on their reserve to meet an increased demand for energy. As osmolarity continued to increase, there was an inhibition of this adaptive mechanism. This, coupled with the reported loss of IGFBP-3 with increasing osmolarity, suggests that IGFBP-3 is an important mediator of corneal epithelial cell stress responses whereby the increase in IGFBP-3 in response to mild or moderate stress is protective. This protective effect is lost in the presence of a substantial stress that downregulates IGFBP-3, leading to a loss of cell viability. Moreover, the measured changes in IGFBP-3 levels were not restricted to corneal epithelial cells, but were evident in conjunctival cells, which are also subject to chronic hyperosmolar stress in dry eye disease. 

A surprising feature of this model was the corresponding decrease in the extracellular acidification rate, a surrogate marker for glycolysis. We have previously shown that growth factor deprivation decreases glycolysis and that this is associated an increase in respiration [43]. However, if the stress is prolonged until mitochondrial respiration decreases due to robust depolarization, glycolysis increases to sustain the cell. Given the high levels of glycogen in the corneal epithelium, the increase in glycolysis is likely due to enhanced glycogenolysis. In contrast to this, we found that hyperosmolar stress decreased both respiration and glycolysis. This occurred in concert with a reduction in protein translation. Thus, in the absence of IGFBP-3, both glycolysis and respiration are suppressed. This was associated with robust mitochondrial fission, as seen by electron microscopy, and the loss of mtDNA. Since fission is essential for mitophagy to occur and attempt to repair damaged mitochondria, these findings would suggest an induction of mitophagy [46]. This is supported by our prior report showing that the loss of IGFBP-3 promotes mitophagy in these same epithelial cells [42]. An unchecked increase in mitophagy can lead to apoptotic cell death, the latter of which has been shown by other studies to occur in response to hyperosmolar stress and in DED [47].

The principal function for IGFBP-3 is to bind IGF-1, inhibiting the IGF-1-mediated activation of IGF-1R. In addition to these IGF-1-dependent effects, IGFBP-3 has also been shown to have IGF-1-independent functions. In the corneal epithelium, we have previously reported that IGFBP-3 mediates the intracellular trafficking of the IGF-1R/INSR hybrid (Hybrid-R) in the absence of IGF-1 [44,48,49]. This occurs through IGFBP-3-mediated SUMOylation of IGF-1R. Here, again, we report on the capacity of IGFBP-3 to induce intracellular trafficking, our second key finding. More specifically, we show that IGFBP-3 drives the mitochondrial localization of IGF-1R. The increase in mitochondrial IGF-1R occurs early in the stress response and was readily detectable by 2 h, as opposed to later changes that we documented after 24 h of hyperosmolar stress. The finding of a mitochondrial-localized IGF-1R during hyperosmolar stress is consistent with our prior work, showing IGF-1R, INSR and EGFR all localizing to the outer mitochondrial membrane [43]. 

With respect to INSR, we previously documented the novel interaction between INSR and the voltage-dependent anion channel (VDAC) [43]. The disruption of this interaction led to mitochondrial instability, depolarization and fragmentation. Since IGFBP-3 induces the shift of IGF-1R to mitochondria during the early stages of hyperosmolar stress, we speculate that it may play an important role in mitochondrial stabilization, while the cell redistributes resources to facilitate metabolic adaptation. This finding is further supported by work showing that IGF-1R functions to maintain mitochondrial integrity and prevent apoptosis [50]. This occurs through the canonical IGF-1/IGF-1R signaling pathway. The ability of IGF-1R to physically stabilize mitochondria and promote survival during stress reflects a previously unrecognized IGF-1-independent mechanism. Mitochondrial-localized IGF-1R was also evident in conjunctival epithelial cells, indicating this is not cornea-specific and may represent a generalized mechanism. 

In contrast to these early changes, after 24 h of hyperosmolar stress, there was a decrease in intra- and extracellular levels of IGFBP-3. This corresponded to a decrease in puromycin-labeled proteins, confirming a hyperosmolar-mediated decrease in protein translation. Co-treatment with IGFBP-3 not only maintained protein translation at an isotonic level, but increased levels of IGFBP-3 in the insoluble nucleus. In our ongoing mouse studies, we similarly found that knockdown of IGFBP-3 using siRNA oligonucleotides administered by subconjunctival injection, also triggered the translocation of IGFBP-3 to the nucleus (unpublished data). The nuclear role of IGFBP-3 in gene transcription has been previously reported [51,52]. Given the close relationship between IGFBP-3 expression and mitochondrial respiration, these data suggest that nuclear IGFBP-3 may promote the expression of genes essential for oxidative phosphorylation. In addition to an IGFPB-3-mediated increase in oxidative phosphorylation, we also found an increase in the outer mitochondrial fusion proteins MFN1 and MFN2, along with an increase in the inner mitochondrial fusion protein, OPA. Of note, OPA also has an integral role in cristae formation [53]. Together, the increase in MFN1 and OPA was shown to drive stress-induced mitochondrial hyperfusion [54]. Indeed, exogenous IGFPB-3 led to the development of hyperfused mitochondria with well-defined cristae and an attenuation of hyperosmolar-mediated loss of mtDNA.

Various types of stress have been shown to disrupt normal mitochondrial dynamics. As is the case illustrated here with hyperosmolarity, mitochondria undergo robust fission. This enables the clearance of damaged mitochondria through mitophagy and is associated with a loss of spare respiratory capacity. In contrast, mitochondria undergo transient hyperfusion in the presence of IGFBP-3. This leads to an increase in respiration and ATP generation, in order to generate the required energy needed to stabilize the cell and mediate metabolic adaptation. In our current model, exogenous IGFBP-3 blocks mitophagy through an increase in fission defects, resulting in hyperfused mitochondria. IGFBP-3 also enhances the lamellar cristae structure, setting the stage for enhanced metabolic capacity. While the mechanism(s) underlying the mitochondrial and metabolic effects of IGFBP-3 are still not well understood, these data point to the clear role of IGFBP-3 as a key mediator of epithelial cell stress responses.

We further illustrated the physiological and translational relevance of IGFBP-3 in vivo, using an aqueous-deficient mouse model of DED. This was accomplished by injecting botulinum toxin into the exorbital lacrimal gland, which in turn, inhibits the release of acetylcholine into the nerve junction, thereby blunting the secretion of tears. Indeed, our data confirm the presence of robust ocular surface damage in the setting of reduced tear production. More important was the finding that tissue levels of IGFBP-3 were similarly reduced in the mouse corneal epithelium. While we have previously shown that IGFBP-3 is present in human tears and is altered in disease, given the small surface area and minute tear volume, the analysis of IGFBP-3 in mouse tear fluid in this study was not feasible [39,40,55]. We did show, however, that the topical administration of IGFBP-3 to the mouse cornea was cytoprotective and significantly decreased corneal surface damage, despite the reduction in tear secretion. Further studies are now needed to evaluate the in vivo effects of IGFBP-3 on mitochondria and metabolic adaptation. In addition, it is unknown whether the protective effects on the mouse cornea are transient in nature or whether prolonged hyperfusion will lead to an accumulation of mitochondrial defects due to impaired mitophagy.

In summary, these findings establish a new functional role for IGFBP-3 in the promotion of mitochondrial hyperfusion and metabolic activity in cells exposed to hyperosmolar stress. These data also support the idea that IGFBP-3 modulates a biphasic response during stress. This includes an early, immediate response by trafficking IGF-1R to the mitochondria to stabilize the organelle, while the nuclear translocation of IGFBP-3 induces metabolic adaptation through increased protein translation, mitochondrial fusion, and oxidative phosphorylation. Future studies will focus on the mechanistic determination of how IGFBP-3 exerts these adaptive effects, whether mitochondrial hyperfusion occurs transiently during metabolic adaptation, or whether it is sustained with potential long-term cellular effects. While current therapeutic interventions for DED are focused on lowering inflammation, these studies could lead to an entirely new class of therapeutics for patients with severe DED and other non-ocular tissues with underlying mitochondrial and metabolic dysfunction. 

## 4. Materials and Methods 

### 4.1. Cell Lines and Culture

Two telomerase immortalized human epithelial cell lines were used in these studies. This includes the human telomerized corneal epithelial (hTCEpi) cell line established and characterized by our laboratory and human conjunctival (HCjE) cells (immortalized with hTERT, mutant Cdk4, and dominant negative p53), which were provided by Drs. Pablo Argüeso (Schepens Eye Research Institute, Harvard Medical School, Boston, MA, USA) and Ajay Sharma (School of Pharmacy, Chapman University, Orange, CA, USA) [56,57,58]. Both cell lines were cultured in serum-free keratinocyte basal media (KBM) with growth factor supplements (KGM, Keratinocyte Growth Medium 2, PromoCell, VWR, Radnor, PA, USA). Due to low levels of calcium in KBM, cultures were further supplemented with a calcium chloride solution (PromoCell, VWR, Radnor, PA, USA) to a final concentration of 0.15 mM. Cultures were maintained at 37 °C with 5% CO_2._ Primary cultures were established from human donor corneas obtained from Tissue Transplant Services at UT Southwestern Medical Center. To accomplish this, human corneal epithelial cells (HCECs) were harvested as previously described [48]. CnT20 cell culture media enriched for progenitor cell culture (Zen Bio, Research Triangle Park, NC, USA) were used for initial cultures. After the first passage, HCECs were transitioned to serum-free KGM media, described above. The osmolarity of the isotonic media was approximately 330 mOsm, as specified by manufacturer data. For hyperosmolarity experiments, hyperosmolar medium was made by the addition of NaCl (Thermo Fisher, St. Louis, MO, USA) to reach the desired osmolarity. Recombinant human (rh)IGFBP-3 (500 ng/mL) was used in select experiments to determine the effects of exogenous IGFBP-3 on all cell types.

### 4.2. Reagents

rhIGFBP-3 was acquired from Sino Biological (Chesterbrook, PA, USA). The lyophilized protein was re-suspended using ultrapure water. Antibodies used for immunoblotting and immunofluorescence included: a rabbit polyclonal anti-IGF-1Rβ #3027, a rabbit monoclonal anti-mitofusion-2 #9482, a rabbit monoclonal anti-mitofusion-1 #14739, a rabbit monoclonal anti-OPA1 #67589, a rabbit monoclonal anti-COX IV #4850, a rabbit polyclonal anti-VDAC1 #4661 (Cell Signaling, Danvers, MA, USA); a mouse monoclonal anti-GAPDH #sc-66163, a mouse monoclonal anti-β-actin #sc-47778 (Santa Cruz Biotechnology, Santa Cruz, CA, USA); and a mouse monoclonal anti-puromycin #MABE343 (EMB Millipore, Burlington, MA, USA). Secondary antibodies used for immunofluorescence were anti-rabbit IgG conjugated to Alexa Fluor 488 and anti-mouse IgG conjugated to Alexa Fluor 555 (Cell Signaling, Danvers, MA, USA). For immunoblotting, secondary antibodies were goat anti-rabbit IgG conjugated to horseradish peroxidase (HRP) #170-6515 and goat anti-mouse conjugated to HRP #170-6516 (Bio-rad, Hercules, CA, USA).

### 4.3. Determination of Cell Number

hTCEpi cells were seeded in 24-well tissue culture plates in KGM at 70% confluence and allowed to adhere overnight. Media were then removed and replaced with isotonic KBM or KBM supplemented with NaCl to a final concentration of 450 mOsm with or without rhIGFBP-3 and cultured for an additional 6 or 24 h. Cell counts and images were captured using a Celigo imaging cytometer (Nexcelom, Lawrence, MA, USA). Cell count experiments were performed in quadruplicate and repeated a minimum of two additional times.

### 4.4. Cell Cycle Assay 

Cell proliferation was measured by seeding hTCEpi cells in a 24-well plate in KGM. Following this, media were replaced with isotonic KBM or KBM supplemented with NaCl to 450 mOsm with or without 500 ng/mL rhIGFBP-3. Cells were incubated in these treatments for 6 or 24 h, as indicated. At the appropriate time points, cells were washed with phosphate-buffered saline and fixed for 15 min with ice-cold ethanol. After fixation, cells were stained with propidium iodide (Thermo Fisher, St. Louis, MO, USA) and imaged using a Celigo imaging cytometer (Nexcelom, Lawrence, MA, USA). Each experiment was repeated a minimum of two additional times.

### 4.5. Enzyme-Linked Immunoassay (ELISA)

Cells were plated using KGM in six-well culture dishes and allowed to adhere overnight. Media were removed and cells were treated with KBM or KBM 450 mOsm media with or without rhIGFBP-3 for 24 h. Media were collected and concentrated using protein concentrators containing a polyethersulfone membrane (3K MWCO; Millipore, Burlington, MA, USA). Cell lysates were harvested using buffer containing 50 mM Tris-HCl pH 7.5, 150 mM NaCl, 1% Triton X-100, 1 mM EDTA, and a protease and phosphatase inhibitor cocktail (Thermo Fisher, Rockford, IL, USA). Whole-cell lysates were kept on ice and vortexed intermittently for thirty minutes. Protein concentration was measured using a Qubit 3.0 Fluorometer (Thermo Fisher, Waltham, MA, USA). A human IGFBP-3 Quantikine enzyme-linked immunoassay (ELISA, R&D systems, Minneapolis, MN, USA) was used to analyze IGFBP-3 levels in conditioned media and whole-cell lysates. Measurements were obtained using a BioTek Synergy 2 Multi-Mode Microplate Reader (Thermo Fisher, Waltham, MA, USA) All samples were run in triplicate and repeated a minimum of two additional times.

### 4.6. Real-Time Metabolic Studies

A Seahorse Metabolic Analyzer XFp (Agilent Technologies, Santa Clara, CA, USA) was used to acquire real-time measurements of cellular oxygen consumption rate (OCR) and intracellular acidification rate (ECAR). hTCEpi cells were seeded into Seahorse XFp miniplates and incubated overnight at 37 °C, 5% CO_2_. The media were then removed, and cells were treated with KBM (basal) or KBM with increasing osmolarity levels (375, 400, or 450 mOsm) for another 24 h at 37 °C, 5% CO_2_. After 24 h, the media were removed and Seahorse XF base medium containing 1 mM pyruvate, 2 mM glutamine, and 10 mM glucose (pH 7.4) was added. Plates were then incubated at 37 °C in a non-CO_2_ incubator for 1 h. Mitochondrial metabolism was analyzed using a Seahorse XFp Cell Mito Stress Test Kit (Agilent Technologies, Santa Clara, CA, USA). During the test, measurements were obtained every 6 min for a total of 94 min. At 20 min, 10 μM oligomycin was added to inhibit ATP synthase. This was followed by an injection of 10 μM carbonyl cyanide 4-(trifluoromethoxy) phenylhydrazone (FCCP) at 50 min, in order to uncouple the proton gradient and allow for maximal respiration. Lastly, an injection of rotenone and antimycin A was added at 80 min, which inhibited complex 1 and 3 of the electron transport chain, thereby disabling mitochondrial respiration, in order to quantify non-mitochondrial respiration. All data were analyzed using the manufacturers’ Wave software, version 2.3.0. The following equations were utilized: spare respiratory capacity = (maximal respiration)/(basal respiration) × 100; coupling efficiency = (ATP-linked respiration rate)/(basal respiration rate) × 100. All six wells were used as technical replicates for each tech condition. The entire experiment was repeated a minimum of two additional times. Immediately after completing the assay, all wells were normalized for cell number using a Celigo Image Cytometer (Nexcelom Bioscience, Lawrence, MA, USA). An Amplex Red Enzyme Assay (Thermo Fisher, Rockford, I) was further used to measure H_2_O_2_ production. This is due to the production of fluorescent resorufin when it reacts with H_2_O_2_. Briefly, hTCEpi cells were seeded onto a 96-black-well plate and allowed to adhere overnight. Media were then changed, and cells were treated for 24 h in isotonic KBM or KBM with increased osmolarity (450 mOsm) with or without rhIGFBP-3. Fluorescence was measured at excitation/emission maxima of 530/590 nm on a Cellometer K2 Fluorescent Viability Cell Counter (Nexcelom, Lawrence, MA, USA). All assays were conducted in triplicate and repeated a minimum of two additional times.

### 4.7. Transmission Electron Microscopy (TEM)

hTCEpi cells were seeded onto 35 mm glass-bottom dishes (MatTek Corporation, Ashland, MA, USA) containing KGM and allowed to adhere overnight. Cells were treated for 24 h in KBM with or without rhIGFBP-3. Following treatment, 2.5% glutaraldehyde/0.1 M cacodylate buffer pH 7.4 was added to the cells for 15 min at room temperature to allow for fixation. Next, cells were washed three times for five minutes with 0.1 M sodium cacodylate buffer. Following washing, cells were post-fixed in 1% osmium tetroxide and 0.8% K_3_[Fe(CN_6_)] in 0.1 M sodium cacodylate buffer for 1 h at room temperature and then rinsed with water. Cells were next stained overnight en bloc with 2% aqueous uranyl acetate and then washed with water. Cells were then dehydrated through exposure to increasing concentrations of ethanol and infiltrated with embed-812 resin and polymerized at 60 °C overnight. Once embedded, cell blocks were sectioned on a Leica Ultracut UCT [7] ultramicrotome (Leica Microsystems, Heidelberg, Germany) using a diamond knife (Diatome, Hatfield, PA, USA) and positioned on copper grids. Finally, samples on copper grids were post-stained with 2% uranyl acetate in water and lead citrate. Imaging was performed using a JEOL 1400 Plus (JEOL) equipped with a LaB_6_ source using a voltage of 120 kV.

### 4.8. Polymerase Chain Reaction

Cells were plated using KGM in six-well culture dishes and allowed to adhere overnight. Media were removed, and cells were treated with KBM or KBM 450 mOsm media with or without rhIGFBP-3 for 24 h. Following treatment, a RNeasy kit (Qiagen, Germantown, MD, USA) was used to extract RNA according to the manufacturer protocol. A gDNA Wipeout Buffer eliminated residual genomic DNA (Qiagen, Germantown, MD, USA). A Nanodrop One^C^ (Thermo Fisher, Rockford, IL, USA) was used to quantify the level of RNA. A QuantiTect Reverse Transcription Kit (Qiagen, Germantown, MD, USA) was used to reverse transcribe 1 μg mRNA into cDNA. A QuantiFast SYBR Green PCR kit (Qiagen, Germantown, MD, USA) was used to perform 40 cycles of real-time PCR on a QuantStudio 6 Flex Real Time PCR machine (Applied Biosystems, Foster City, CA, USA). For real-time PCR, 100 ng cDNA was amplified by using 1 µM of each QuantiTect Primer Assay (Qiagen, Germantown, MD, USA) for Hs_EIF2AK1 (QT01018920), Hs_PTGS2 (QT00040586) and Hs_LOC100128596 (QT02432626). These correspond to a mitochondrial encoded gene, Cyclooxygenase 2 (COX2), which was analyzed along with two nuclear encoded genes, Eukaryotic Initiation Factor 2 (EIF2) and NADH Dehydrogenase. Levels of COX2 were normalized to each of the two nuclear encoded genes for normalization. Total reaction volume was 10 µL. Samples were plated in triplicate for all experiments. Water was used as a no-template control. All controls were performed in parallel. Experiments were repeated a minimum of two additional times. Data were analyzed using the 2-ΔΔCT method. 

### 4.9. siRNA Knockdown of IGF-1R

hTCEpi cells were seeded at 50–60% confluence into six-well plates and allowed to adhere overnight. Cells were then transfected with double-stranded inhibitory RNA oligonucleotides to IGF-1R or IGFBP-3 (FlexiTube GeneSolution, IGF-1R #GS3480, Qiagen, Germantown, MD, USA), using Lipofectamine RNAiMAX (Invitrogen, Carlsbad, CA, USA) in antibiotic-free KGM. An amount of 12 pmol of siRNA oligonucleotides targeting IGF-1R or non-targeting controls were added to 100 µL KGM and incubated at room temperature for five minutes. The siRNA was combined with 2 µL Lipofectamine diluted in 100 µL KGM. This mixture was incubated for fifteen minutes at room temperature. Next, the transfection mixture was added to hTCEpi cells containing 1 mL of KGM media. Samples were then incubated for 24 h prior to treatment. Transfection media were removed, and cells were cultured in 450 mOsm KBM for another 24 h, with IGFBP-3 confirming the specificity of the antibody for IGF-1R in the mitochondrial fraction. Allstars negative control siRNA was used as the non-targeting control (Qiagen, Germantown, MD, USA).

### 4.10. Sodium Dodecyl Sulfate Polyacrylamide Gel Electrophoresis and Immunoblotting 

For immunoblotting of whole-cell lysates, a lysis buffer containing 50 mM Tris-HCl pH 7.5, 150 mM NaCl, 1% Triton X-100, 1 mM EDTA, and a protease and phosphatase inhibitor cocktail (Thermo Fisher, Rockford, IL, USA) was used to directly lyse adherent epithelial cells in 6-well culture plates. Samples were kept on ice and vortexed intermittently for 30 min, followed by centrifugation for 5 min at 12,000 rpm at 4 °C (BioRad, Hercules, CA, USA). After centrifugation, the supernatants were collected and protein concentration was quantified using a Qubit 3.0 Fluorometer (Thermo Fisher, Rockford, IL, USA). A 2× sample buffer pH 6.8 containing 65.8 mM Tris-HCl, 26.3% (*w*/*v*) glycerol, 2.1% SDS, 5.0% β-mercaptoethanol and 0.01% bromophenol blue was added to the sample, which was then boiled for five minutes (Bio-rad, Hercules, CA, USA). Next, boiled samples were electrophoresed through a 4–15% precast linear gradient polyacrylamide gel (Bio-rad, Hercules, CA, USA). Once electrophoresis was complete, gels were transferred onto a polyvinyl difluoride (PVDF) membrane (Millipore, Temecula, CA, USA). Membranes were then blocked with 5% non-fat milk for one hour at room temperature (Bio-rad, Hercules, CA, USA). After blocking, membranes were washed three times for five minutes with PBS. Membranes were incubated overnight in primary antibody at 4 °C and then washed again three times for five minutes. Membranes were incubated with secondary antibody at room temperature for one hour. Secondary antibody was either anti-mouse or anti-rabbit secondary antibody (Santa Cruz, CA, USA). An Amersham Imager 600 (Amersham Biosciences, Piscataway, NJ, USA) was used to image the protein present on the membrane with ECL Plus Prime Detection Reagent (Amersham Biosciences, Piscataway, NJ, USA). β-actin or GAPDH were used as loading controls for normalization. ImageQuant TL Toolbox v8.1 software was used to analyze and quantify proteins (Amersham Bioscience, Piscataway, NJ, USA). To measure global protein translation, a SUnSET Assay was used. hTCEpi cells were seeded into a six-well plate at 70% confluence and allowed to adhere overnight. The media were then replaced with isotonic KBM (basal) or 450 mOsm KBM with or without rhIGFBP-3. Two sets of each group were treated, and cells were cultured for 22 h under these conditions. At 22 h, one set from each group was treated with cycloheximide solution (Sigma, St. Louis, MO, USA). All groups were then immediately treated with puromycin dihydrochloride (Sigma, St. Louis, MO, USA). Cells were cultured for an additional two hours. Cells were then lysed and blotted as described above.

### 4.11. Nuclear and Mitochondrial Fractionation with Immunoblotting

For experiments quantifying the intracellular localization of IGFBP-3 and IGF-1R, hTCEpi cells were subjected to subcellular fractionation. Trypsin-ethylenediaminetetraacetic acid (EDTA, Gibco, St. Louis, MO, USA) was used to dissociate adherent cells after 2 or 24 h of treatment in isotonic KBM or 450mOsm KBM with or without IGFBP-3. Cells were then centrifuged and washed with PBS prior to use. To isolate the mitochondrial fraction, a mitochondrial fractionation kit (Thermo Fisher, Rockford, IL, USA) was used. A nuclear fractionation kit (Thermo Fisher, Rockford, IL, USA) was used to separate the cytoplasmic, insoluble and soluble nuclear fractions. Fractions were boiled with 2× sample buffer and electrophoresed through a 4–15% polyacrylamide gel (BioRad, Hercules, CA, USA). Immunoblotting was performed as described above. A voltage-dependent anion channel (VDAC) was used as a mitochondrial control. Histone H3 was used as a control for the insoluble nuclear fraction and SP1 for the soluble nuclear fractionation.

### 4.12. Botulinum-Toxin-B-Induced Dry Eye Disease Mouse Model

A total of 104 mice were used in this study. All mice were C57BL6 Type N strain aged 6 to 8 weeks (Charles River, Wilmington, MA, USA). An aqueous-deficient dry eye model was used according to a previously reported method [59,60]. All mice for these studies were housed at a relatively stable temperature (21–24 °C) and humidity. A sterile 33G needle was used to inject the lacrimal gland with botulinum toxin-B (Botox, 20 mU in 0.05 mL 0.9% saline) or the vehicle control (0.05 mL of 0.9% saline). Only the right eye was used for these experiments due to the potential for sympathetic responses between eyes. Mice were randomly assigned to the Botox or control group and there were an equal number of male and females in each group. All mice were screened prior to injection for ocular surface abnormalities. To determine the effect of topical treatment of rhIGFBP-3 in DED, 10 μL of a solution containing 500 ng/mL of rhIGFBP-3 was applied on the surface of the eye. Either rhIGFBP-3 or the vehicle control (sterile saline) were applied twice a day on alternating days for the final seven days prior to tissue collection and analysis.

### 4.13. Clinical Measurements

To quantify ocular surface damage, fluorescein staining was performed at Day 0, 7, 14, and 28 to determine the severity of DED. Fluorescein sodium ophthalmic strips (1 mg, BioGlo, Scottsdale, AZ, USA) were used for these experiments. Examination of the ocular surface was performed using a slit-lamp biomicroscope with a cobalt blue light and yellow Wratten filter. Photographs of each mouse were obtained using a Sony Cyber Shot camera (New York, NY, USA) fitted to the slit-lamp eye piece. For fluorescein grading, the eye was subdivided into five quadrants that were scored separately by a blinded observer. Each quadrant was scored using a scale of 0–3 with 0 indicating no staining and 3 indicating severe ocular surface damage [61]. The total score was the sum of the score from all five quadrants. A phenol red thread test was used to determine aqueous tear production (Zone-Quick, Oasis, CA, USA). The tip of the thread was placed in the lateral canthus of the right eye for 15 s. The length of thread that had turned red was measured using a micron ruler.

### 4.14. Immunofluorescence

Mouse corneas and hTCEpi cells were used for immunofluorescent assays. For the in vitro studies, hTCEpi cells were seeded onto 35 mm glass-bottom dishes (MatTek Corporation, Ashland, MA, USA). siRNA was used to knockdown IGFBP-3 as described and cells were incubated for 24 h. Cells were then cultured in KBM (basal) with or without rhIGFBP-3 for an additional 24 h. After, cells were washed twice with cold PBS and fixed in 1% paraformaldehyde (Electron Microscopy Sciences, Fort Washington, PA, USA) in PBS for 10 min. Following this, cells were washed with PBS and then permeabilized in 0.1% Triton X-100 in PBS for 10 min. Samples were then washed three times for five minutes with PBS. Next, cells were blocked using 0.5% bovine serum albumin (Sigma, St. Louis, MO, USA) in PBS for 30 min. Cells were incubated overnight at 4 °C using primary antibodies directed against MFN1, MFN2, or IGFBP-3 diluted in 0.1% bovine serum albumin (Sigma, St. Louis, MO, USA). After, each sample was washed in PBS and stained with an anti-rabbit IgG conjugated to Alexa Fluor 488 for 1 h at room temperature. Prolong gold anti-fade reagent containing DAPI was used for mounting and nuclear staining (Invitrogen, Carlsbad, CA, USA). In order to analyze IGFBP-3 expression in the mouse corneal epithelium, we performed both ELISA (detailed earlier) and immunofluorescence. For the latter, whole-mouse globes were excised, fixed in 1% paraformaldehyde, and embedded in Tissue-Plus™ O.C.T. Compound Tissue-Plus™ embedding medium (Thermo Fischer, Waltham, MA, USA) and frozen in a −80 °C freezer. Cryostat-sectioned tissues were washed three times with PBS for five minutes. Following this, tissue sections were rewashed with PBS and permeabilized using ice-cold acetone. After washing, tissue sections were blocked with 0.5% BSA in PBS and then stained using primary and secondary antibodies as described above. Cells were imaged on a Leica SP8 laser scanning confocal microscope (Leica Microsystems, Heidelberg, Germany) using a 63× oil objective. In order to prevent spectral crosstalk between channels, images were sequentially scanned.

### 4.15. Statistics

All data are presented as mean ± standard deviations. For the comparison between two groups, a Student’s t-test was utilized. For comparisons between more than two groups, a one-way ANOVA was used. For ANOVA analysis, an appropriate post hoc multiple comparison test was performed. Statistical significance was set at *p* < 0.05.

## Data Availability

The authors confirm that the data supporting the findings of this study are available within the article [and/or] its Supplmentary Materials.

## Supplementary Materials

The following supporting information can be downloaded at: https://www.mdpi.com/article/10.3390/ijms23074066/s1.
