# Supplementary material for: IGFBP-3 Regulates Mitochondrial Hyperfusion and Metabolic Activity in Ocular Surface Epithelia during Hyperosmolar Stress
Source: Int J Mol Sci. 2022 Apr 6;23(7):4066. doi: 10.3390/ijms23074066 (PMC9000157; doi:10.3390/ijms23074066)

Supplemental Data

Supplemental Figure S1: Hyperosmolarity alters IGFBP-3 levels. Cells were treated with KBM with increasing levels of salt for 24 hours. Intracellular and extracellular levels of IGFBP-3 were measured using ELISA. (**A&B**) Intracellular (**A**) and extracellular (**B**) levels of IGFBP-3 in HCjEC. Although not significant, there was a trend towards an increase in IGFBP-3 expression and secretion in HCjEC at 375 mOsm. Similar to the other cell types, increasing levels of hyperosmolar stress decreased IGFBP-3 expression levels (*P<0.001). (**C&D)** Intracellular (**C**) and extracellular (**D**) levels of IGFBP-3 in HCECs. Consistent with hTCEpi cells, HCECs showed a significant increase in IGFBP-3 expression and secretion at 375 mOsm, followed by a decrease at higher levels of salt (**P<0.01 significantly different from all other groups; ***P<0.001, significantly different from all groups except each other). Graphs representative of a single experiment repeated 3 times. Data presented as mean ± standard deviation, one-way ANOVA, SNK multiple comparison test.

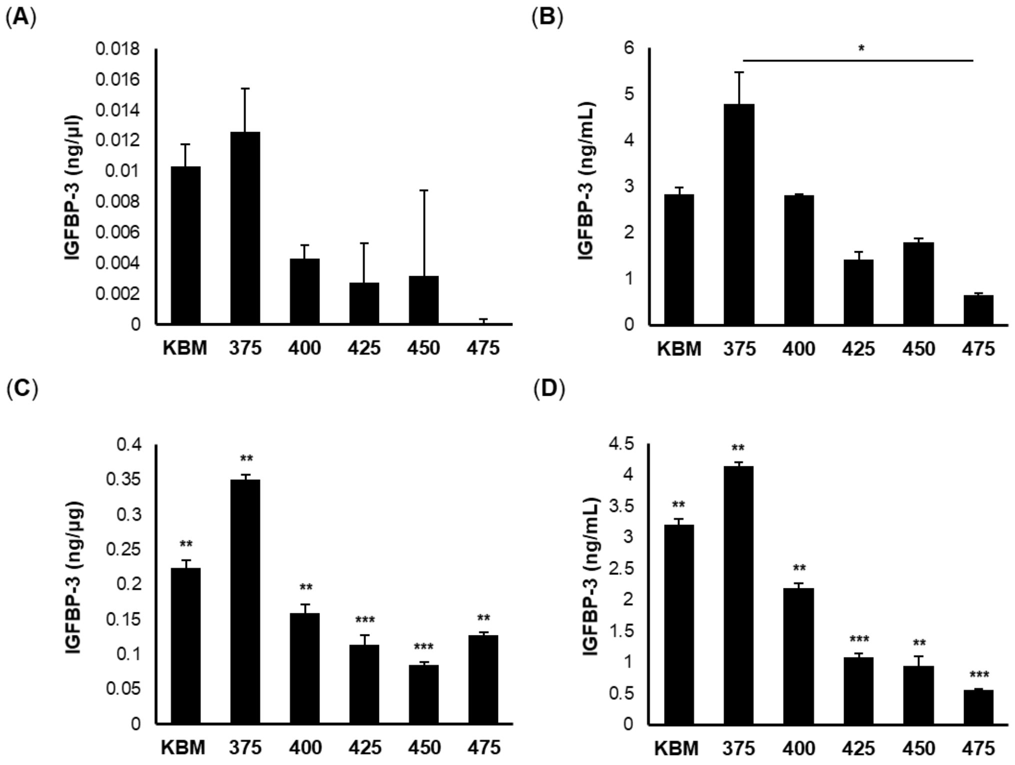

Supplemental Data

Supplemental Figure S2: IGFBP-3 alters mitochondrial bioenergetics in cells exposed to hyperosmolar stress. hTCEpi cells were cultured in isotonic KBM and supplemented with increasing levels of salt for 24 hours. Cells in 450 mOsm KBM were cultured with or without 500 ng/mL rhIGFBP-3. (**A**) Basal respiration was decreased at 450 mOsm compared to the test and control groups. (**B&C**) There were no differences in (**B**) non-mitochondrial oxygen consumption or (**C**) proton leak. (**D&E**) Both (**D**) maximal respiration and (**E**) coupling efficiency were decreased at 450 mOsm compared to the test and control groups. Graphs representative of a single experiment repeated 3 times. Data presented as mean ± standard deviation, one-way ANOVA, SNK multiple comparison test.

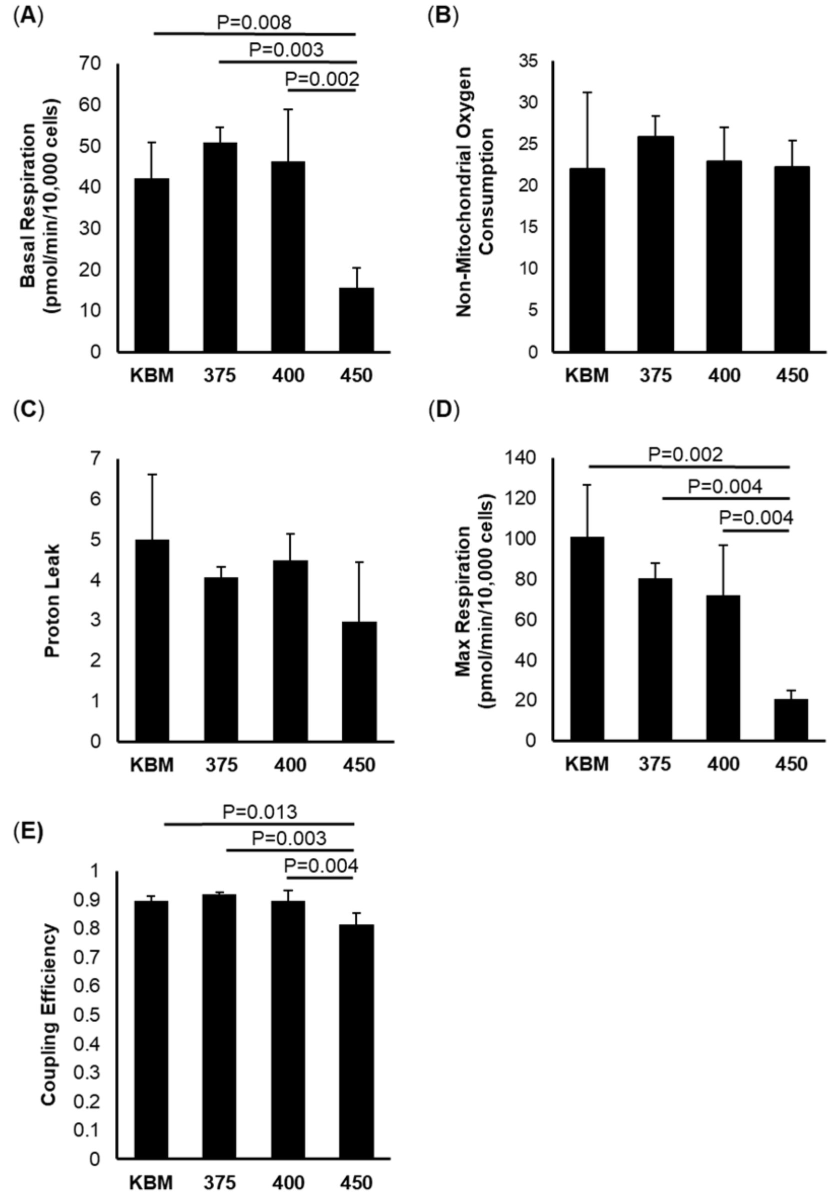

Supplemental Data

Supplemental Figure S3: IGFBP-3 mediates mitochondrial trafficking of IGF-1R. hTCEpi cells were transfected with siRNA oligonucleotides targeting IGFBP-3. Non-targeting oligonucleotides were used as a control. (**A**) hTCEpi cells were separated into cytosolic and mitochondrial fractions and subject to immunoblotting for IGF-1R. VDAC was used as a control for the mitochondrial fraction, GAPDH for cytosol. IGFBP-3 knockdown cells were treated with or without rhIGFBP-3. (**B**) While IGF-1R showed a trend towards a reduction in mitochondrial expression in IGFBP-3 knockdown cells, it was not was not significantly different. In contrast, treatment with rhIGFBP-3 increased mitochondrial levels of IGF-1R (mtIGF-1R). Immunoblots representative of 3 repeated experiments. Data presented as mean ± standard deviation, one-way ANOVA, SNK multiple comparison test.

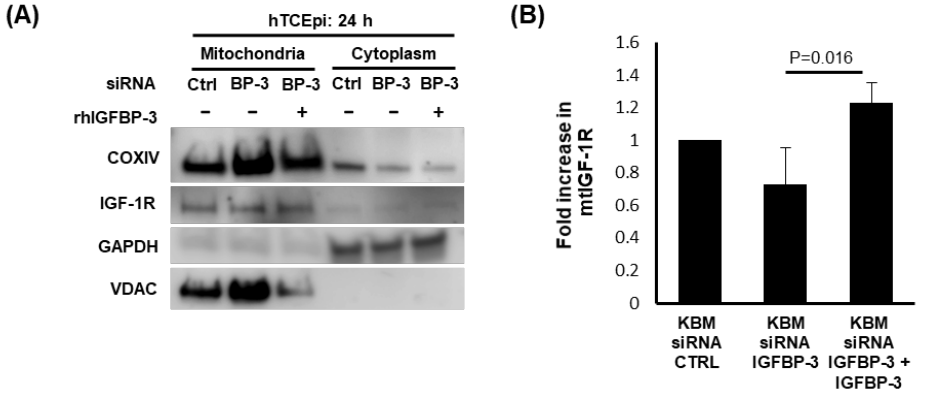

Supplemental Data

Supplemental Figure S4: DED increases ocular surface damage. The cornea was subdivided into five quadrants for grading. Corneal staining using fluorescein was graded on a scale of 0-3. (**A**) Schematic demonstrating the five quadrants the cornea. (**B-F**) There was an increase in corneal staining in botox treated eyes at all time points examined in (**B**) quadrant 1, (**C**) quadrant 2, (**D**) quadrant 3, (**E**) quadrant 4, and (**F**) quadrant 5. Representative images of corneal fluorescein staining shown, N=10 mice/group on day 7, N=10 mice/group on day 14, and N=9 mice per group on day 28. Data presented as mean ± standard deviation, Student's t-test comparing the botox treated eye to control at each time point.

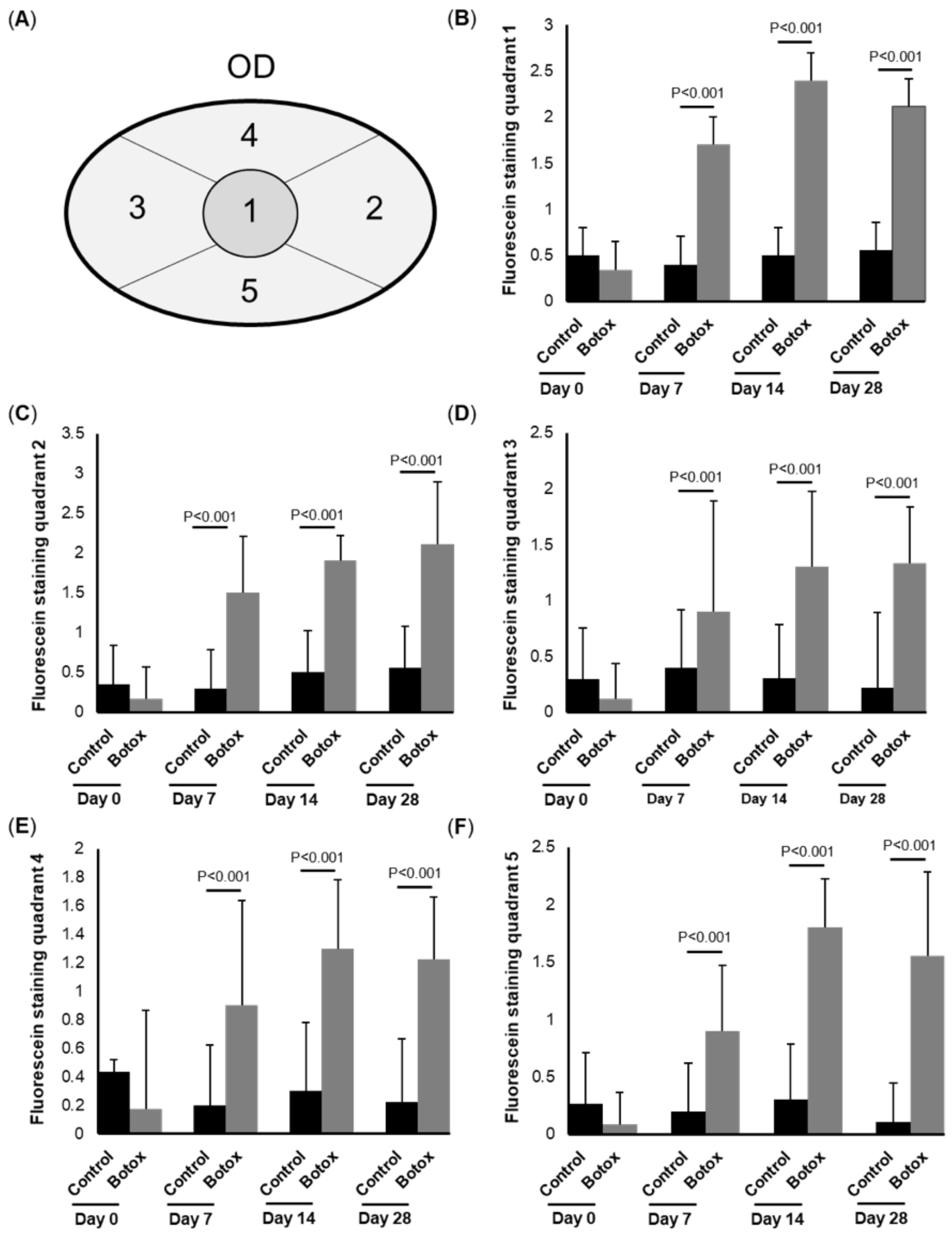

Supplemental Data

Supplemental Figure S5: Treatment with rhIGFBP-3 improves corneal staining. Using our botox model, we examined corneal staining across all five quadrants in the cornea. Corneas were examined using a slit lamp biomicroscope at baseline and again on days 7, 14, and 28 post-injection. (**A-E**) Corneal staining was decreased by topical treatment with rhIGFBP-3 in (A) quadrant 1, (B) quadrant 2, (C) quadrant 3, (D) quadrant 4, and (E) quadrant 5. All reductions were significant except for quadrant 4, which is a covered region under the upper eyelid. Data presented as mean ± standard deviation, Student's t-test, N=8 mice per group.

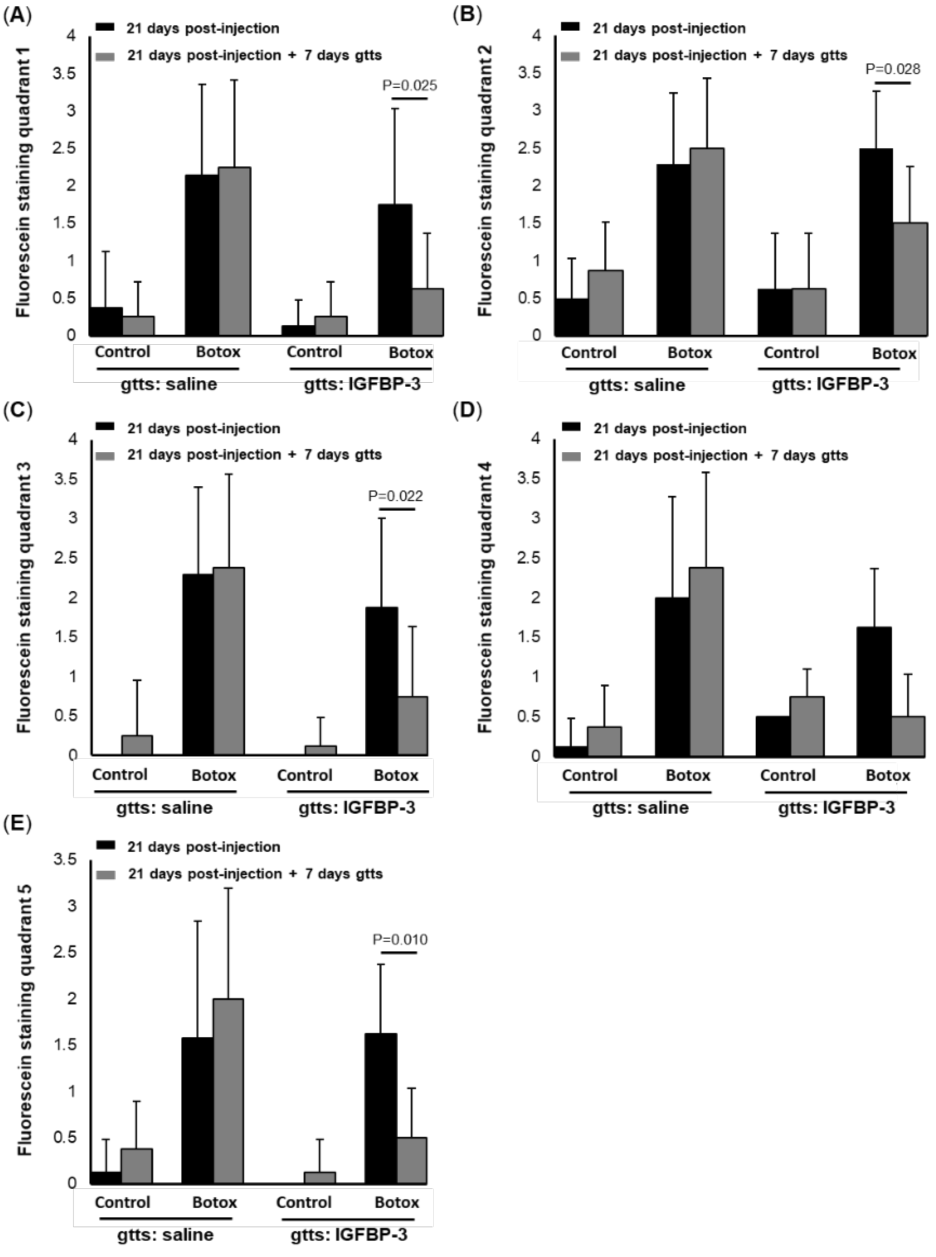

Supplement: Supplementary file 1 [file ijms-23-04066-s001.zip › ijms-1601242-supplementary.pdf]
